# The effectiveness of Kami Guibi-tang for cognitive impairment patients: A retrospective chart review

**DOI:** 10.1016/j.heliyon.2023.e23615

**Published:** 2023-12-15

**Authors:** Tae-Bin Yim, Gyu-Ri Jeon, Hye-Jin Lee, Kyeong-Hwa Lee, Hye-Min Heo, Han-Gyul Lee, Seungwon Kwon, Seung-Yeon Cho, Seong-Uk Park, Woo-Sang Jung, Sang-Kwan Moon, Chang-Nam Ko, Jung-Mi Park

**Affiliations:** a Stroke and Neurological Disorders Center, Kyung Hee University College of Korean Medicine, Kyung Hee University Hospital at Gangdong, Seoul, South Korea; b Department of Clinical Korean Medicine, Graduate School, Kyung Hee University, Seoul, South Korea; c Department of Cardiology and Neurology, College of Korean Medicine, Kyung Hee University, Seoul, South Korea; d Department of Cardiology and Neurology, Kyung Hee University College of Korean Medicine, Kyung Hee University Medical Center, Seoul, South Korea

**Keywords:** Alzheimer's disease, Herbal medicine, Kami Guibi-tang, Mild cognitive impairment, Subjective cognitive decline, Vascular dementia

## Abstract

**Background:**

and Purpose: The number of patients with cognitive impairment is increasing worldwide. Therapeutic drugs that slow disease progression are being developed; however, further research is required. This study investigated the effects of Kami Guibi-tang on patients with various types of cognitive decline.

**Methods:**

This study was a single-center, retrospective chart review of patients who visited KyungHee University Hospital at Gangdong from January 2015 to March 2022. The study included participants who took Kami Guibi-tang for more than 90 days and were assessed on the Korean version Mini-Mental State Examination (MMSE-K) scores before and after treatment. Participants who received other liquid herbal medicines during the treatment were excluded. The outcome of interest was changed scores in MMSE-K and Short form of Geriatric Depression Scale (S-GDS).

**Results:**

A total of 31 participants were included. The total MMSE-K score significantly increased with time and showed a significant increase at 3 and 9 months compared with baseline. Among the MMSE-K subscores, the orientation subscore showed a significant increase at three months compared with baseline, and the attention and calculation subscore showed a significant increase with time. In addition, four participants with vascular dementia showed a significant increase in the total MMSE-K score over time and a significant increase after 9 months compared with baseline. The S-GDS score in 31 participants showed a significant decrease with time and at all time points compared with baseline.

**Conclusions:**

Kami Guibi-tang may improve cognitive function in patients with cognitive decline.

## 1 Introduction

Recently, as the average human life expectancy has increased, the elderly population has been rapidly increasing; accordingly, the number of patients with cognitive impairment is also increasing. Therefore, an increasing number of patients visit hospitals due to concerns about the transition to dementia and the resulting deterioration in the quality of life of patients and their dependents. In Korea, the proportion of senior citizens aged 65 years and older is rapidly increasing, with 7.2 % in 2000, 14 % in 2017, and 16.7 % of the total population in 2021 [1,2]. As the elderly population increases, the number of dementia patients has increased by approximately 50,000 every year since 2017, reaching an estimated 892,000 dementia patients aged 65 years or older in 2021. The estimated prevalence of dementia has been reported to be 10.33 % [1].

Dementia refers to a loss of memory, language, problem solving and other thinking abilities that is severe enough to interfere with daily life. Diseases that manifest these symptoms include Alzheimer's disease (AD), vascular dementia (VD), frontal temporal lobe degeneration, and Levisoche dementia [2]. Vascular cognitive impairment refers to any cognitive impairments caused by cerebrovascular disease [3]. Pre-stage dementia includes mild cognitive impairment (MCI) and subjective cognitive decline (SCD). MCI refers to a state in which cognitive impairment is objectively shown but daily life ability is maintained, unlike dementia, while SCD refers to a subjective decrease in cognitive function, although there is no objective cognitive impairment [4].

Currently, there are no drugs that can cure dementia. Aducanumab has recently been approved by the U.S. Food and Drug Administration (FDA), but its effectiveness is still controversial [5]. Lecanemab (Leqembi), which slows disease progression, has also been approved by the U.S. FDA. In Korea, acetylcholine-degrading enzyme inhibitors such as donepezil, rivastigmin, galantamine, and memantine, an N-methyl-d-aspartate (NMDA) receptor antagonist, are used. The standard treatment for dementia is also used for MCI, but the effect is not clear, and the side effects cannot be ignored [6]. Therefore, there is an emerging need for complementary and alternative medicines.

Kami Guibi-tang (KGT) is a traditional herbal formula composed of 15 herbs and plants (Table 1) that has been mainly used to treat insomnia, mental anxiety, and nervousness [7], and a number of clinical and experimental studies have reported improvements in memory. Experimental studies have reported improvement in object recognition memory in a mouse model of AD [8] and the protective effect of delayed neural death in CA1 pyramidal cells in the hippocampus [9]. In clinical studies, the memory area score of the Seoul Neuropsychological Screening Battery (SNSB-D) increased particularly after KGT administration, in patients with MCI, and a significant improvement in the Clinical Duration Rating Sum of Box (CDR-SB) was reported [10]. In patients with mild AD, the Mini Mental State Examination (MMSE) score was maintained [11] and another study reported significant improvement in the MMSE score [12]. There have also been reports of alcohol-related dementia [13].

**Table 1:** The composition of Kami Guibi-tang (grams).

Herb	Scientific name	Latin name	Kyungbang co. (per 1 pack)	Kracie pharma korea co. (per 1 day)
人蔘	*Panax ginseng* C. A. Meyer	Ginseng Radix	1.0	3.0
白朮	*Atractylodes macrocephala* Koidzumi	Atractylodis Rhizoma Alba	1.0	3.0
茯苓	*Poria cocos* Wolf	Poria Sclerotium	1.0	3.0
黃芪	*Astragalus membranaceus* Bunge	Astragali Radix	1.0	2.0
龍眼肉	*Dimocarpus longan* Loureiro	Longanae Arillus	1.0	3.0
酸棗仁	*Zizyphus jujuba* Miller var. *spinosa* Hu ex H. F. Chou	Zizyphi Semen	1.0	3.0
柴胡	*Bupleurum falcatum* Linné	Bupleuri Radix	1.0	3.0
當歸	*Angelica gigas* Nakai	Angelicae Gigantis Radix	0.67	2.0
遠志	*Polygala tenuifolia* Willdenow	Polygalae Radix	0.67	1.5
梔子	*Gardenia jasminoides* Ellis	Gardeniae Fructus	0.67	2.0
牧丹皮	*Paeonia suffruticosa* Andrews	Moutan Cortex	0.67	–
大棗	*Zizyphus jujuba* Miller var. *inermis* Rehder	Zizyphi Fructus	0.67	1.5
木香	*Aucklandia lappa* Decne	Aucklandiae Radix	0.33	1.0
甘草	*Glycyrrhiza uralensis* Fischer	Glycyrrhizae Radix et Rhizoma	0.33	1.0
生薑	*Zingiber officinale* Roscoe	Zingiberis Rhizoma Recens	0.33	0.5
Suggested Usage			3 times per day	2 times per day

Several clinical studies have examined the effects of KGT on MCI and AD; however, all were limited to conditions that were somewhat removed from the actual medical office environment. In a clinical study of patients with MCI conducted by Shin et al. [10], a significant improvement in CDR-SB and in the memory area of SNSB-D in the KGT group compared with the placebo group was observed, but this was a preliminary study that requires further verification. In addition, clinical studies targeting subjective cognitive decline have not yet been conducted, and there have only been case reports of vascular cognitive impairment [14].

Therefore, in this retrospective study, the clinical effects of KGT on various types of cognitive impairments in patients who visited a single oriental medicine hospital were analyzed and examined comprehensively. As a result, it is anticipated that it will be possible to create a foundation for clinical research on cognitive impairment and assist clinicians in establishing a framework for employing KGT in patients with cognitive impairment.

## 2 Materials and methods

### 2.1 Participants

Patients’ medical records were collected from the Korean Internal Medicine Department at the Stroke and Neurological Disorders Center of Kyunghee University Hospital at Gangdong from January 1, 2015, to March 17, 2022. Those who had taken KGT granules (HH856 - Kyungbang Pharmaceutical Co.; HH846, – Kracie Holdings, Ltd.) for at least 90 days in a row, and had undergone the Korean version of Mini Mental State Examination(MMSE-K) more than once, met the criteria.

Patients were included based on the following criteria: those who underwent the MMSE-K before and after taking KGT granules and a follow-up MMSE-K 3 months ± 1 monthafter the previous evaluation date. Patients were excluded based on the following criteria: receiving both KGT granules and other herbal medicines at the same time; not follow-up MMSE-K at 3 months ± 1 month after the previous evaluation date; and no MMSE-K before and after taking KGT.

### 2.2 Data collection

Data on the following items were collected and retrospectively analyzed from the electronic medical records of participants satisfying the inclusion and exclusion criteria.

Information on each patient's age, sex, impression, and medication history was collected as general characteristics. Total scores on the MMSE-K, Short form of the Geriatric Depression Scale (S-GDS), Neuropsychiatric Inventory Questionnaire (NPI-Q), Instrumental Activities of Daily Living (I-ADL), Clinical Dementia Rating Global Score (CDR-GS), CDR-SB score, and date of each assessment were also collected as clinical characteristics.

#### 2.2.1 Primary index

##### 2.2.1.1 Changes in MMSE-K total score and sub scores

Changes in the MMSE-K total and sub-scores were confirmed after three, six, and nine months based on the score at the initial evaluation (before taking KGT). The MMSE-K is the most widely used cognitive function screening tool and is included in the National Institute of Neurological Communicative Disease and Stroke and Alzheimer's Disease and Related Disorders Association [14]. There were six detailed items: support history; memory registration; memory recall; attention, focus and calculation; language; and comprehension and judgment [15].

#### 2.2.2 Secondary index

##### 2.2.2.1 Changes of MMSE-K total score and sub scores in subtypes

The initial evaluation values of the total MMSE-K score, changes in the scores of the sub-items, and changes at three, six, and nine months were confirmed. The subtypes were categorized as AD, MCI, SCD, and VD.

The subtype classification followed the diagnosis name when the diagnosis of a neurologist was written in the electronic medical record; if there was no record of a diagnosis, it was defined as follows: AD was defined as less than 20 MMSE points according to the moderate-to-severe AD standard [16]. SCD was defined as no objective cognitive impairment; based on previous studies that reported 92 % sensitivity and 91.5 % specificity when the cut-off was set to 24 or 23 points [17], it was set to 24 points or higher. MCI was define as 20 points or more, or less than 24 points on the MMSE. VD was defined as cognitive impairment following a stroke [4].

##### 2.2.2.2 Changes of S-GDS score

We checked the baseline and changes after three months using the S-GDS, a shortened depression scale for evaluating depression symptoms in the elderly that includes questions on memory loss and cognitive impairment, which are the main symptoms associated with depression. The higher the total score out of 15, the more severe the tendency toward depression, and, in general, a score of 8 or higher indicates a high likelihood that the patient is experiencing depression symptoms [18].

#### 2.2.3 Statistical analysis

SPSS Version 18.0 Windows (Chicago, IL, USA) was used for the statistical analysis. For changes in MMSE-K scores by all participants and subtypes, one-way repeated measures analysis of variance (ANOVA) was performed for normally distributed data for changes in scores at 3, 6, and 9 months based on the test value of the initial evaluation, and post-analysis was also confirmed through one-way repeated measures ANOVA with Bonferroni Correction. If a normal distribution was not observed, the change in score over time was analyzed using Friedman's test, and the Wilcoxon signed-rank test was used for post-analysis. When changes in scores over time showed a normal distribution, based on the initial evaluation test values, a one-way reflected-measures ANOVA was performed, and post-analysis was also confirmed through a one-way repeated-measures ANOVA with Bonferroni correction. If a normal distribution was not observed, the change in score over time was analyzed using Friedman's test, and the Wilcoxon signed-rank test was used for post-analysis. Normality was tested using the Kolmogorov-Smirnov test, and the Shapiro-Wilk test was performed at a significance level of p < 0.05. All statistics of this study were expressed as mean ± standard deviation (SD) if normal among continuous variables, median (Q1-Q3) if not normal, and number (%) if categorical variables.

Missing data were replaced with the last observation carried forward (LOCF) imputation method to maintain the sample size and reduce the bias caused by participant attrition.

## 3 Results

**Fig. 1:**
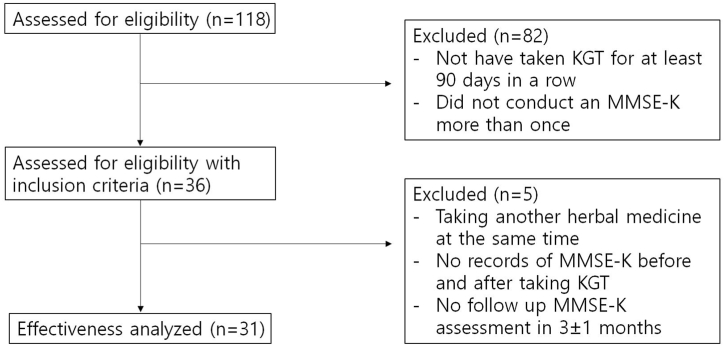
A flow chart of our study.

A total of 36 patients visited the study institution from January 1, 2015, to March 17, 2022, and received KGT for at least 90 consecutive days. Thirty-one patients were included in the study. The remaining five were excluded for the following reasons: MMSE-K evaluation cycle of less than 3 months ± 1 month; no record of MMSE-K evaluation before or after taking KGT; and taking decoction at the same time as KGT (Fig. 1).

### 3.1 Demographic and clinical analysis

#### 3.1.1 General characteristics

A total of 31 participants who met the inclusion and exclusion criteria were analyzed for their general characteristics. The mean age was 69.6 ± 11.2 years, and nine of the patients (29 %) were male. 21 patients (67.7 %) were taking cognitive function-improving drugs such as donepezil, rivastigmine, galantamine, and memantine; brain function-improving drugs such as gliatirin and gliatamine, which can affect brain cognition; and nutritional supplements.

Of the 31 participants, four had AD, 17 had MCI, four had SCD, and six had VD (three with intracerebral hemorrhage, two with cerebral infarction, and on with subarachnoid hemorrhage). The median age of the four AD patients was 64.50 (56.50–68.75) years, and 25 % were male. The mean age of the 17 patients with MCI was 71.2 ± 10.3 years old; 17.6 % were male; and 88.2 % were taking drugs to improve cognitive function. The median age of the four patients with SCD was 67.50 (61.50-28.50) years, of whom 75 % were male and 25 % were taking cognitive function-improving drugs. The median age of the six patients with VD was 70.50 (53.75–84.50) years, of which 33.3 % were male and 50.0 % were taking cognitive function improvement drugs (Table 2). Additionally, the median time of KGT administration from the onset of cerebrovascular disease, which is the cause of VD, was 9 (2–42) months (Table 2).

**Table 2:** Demographic and baseline characteristics of participants.

	Total (n = 31)	AD group (n = 4)	MCI group (n = 17)	SCD group (n = 4)	VD group (n = 6)
Age	69.6 ± 11.2	64.50 (56.50–68.75)	71.2 ± 10.3	67.50 (61.50–82.50)	70.50 (53.75–84.50)
Sex(Male)	9 (29.0 %)	1 (25 %)	3 (17.6 %)	3 (75 %)	2 (33.3 %)
Onset (Months ago)	–	–	–	–	9 (2–42)
Taking medication affecting cognitive function	21 (67.7 %)	4 (100 %)	15 (88.2 %)	1 (25 %)	3 (50.0 %)
MMSE-K Total score	23.32 ± 4.66	16.50 (14.25–19.50)	23.35 ± 4.43	26.00 (25.25–28.25)	26.00 (21.75–29.00)
Orientation	8.06 ± 2.00	6.00 (4.50–6.75)	8.00 (6.00–10.00)	9.50 (9.00–10.00)	10.00 (8.50–10.00)
Registration	2.74 ± 0.68	2.00 (1.00–3.00)	3.00 (3.00–3.00)	3.00 (3.00–3.00)	3.00 (3.00–3.00)
Recall	1.35 ± 1.14	1.00 (0.25–1.75)	1.00 (0.00–2.50)	2.50 (0.50–3.00)	1.50 (0.75–2.00)
Attention & Calculation	2.90 ± 1.92	0.50 (0.00–1.00)	4.00 (1.00–5.00)	3.50 (2.25–4.75)	4.00 (1.00–5.00)
Language	6.39 ± 0.84	5.50 (5.00–6.75)	7.00 (6.00–7.00)	6.50 (6.00–7.00)	7.00 (6.25–7.00)
Comprehension & Judgment	1.90 ± 0.40	2.00 (2.00–2.00)	2.00 (2.00–2.00)	2.00 (2.00–2.00)	2.00 (2.00–2.00)

AD, Alzheimer's disease; MCI, mild cognitive impairment; SCD, subjective cognitive decline; VD, vascular dementia; MMSE-K, Korean version of Mini-Mental State Examination.

Values are presented as mean ± SD, median (Q1-Q3) or number (%).

#### 3.1.2 Clinical characteristics

Before taking KGT, all 31 patients had an average MMSE-K score of 23.32 ± 4.66 points; orientation was 8.06 ± 2.00 points; memory registration 2.74 ± 0.68 points; memory recall was 1.35 ± 1.14 points; attention was 2.90 ± 1.92 points; language function was 6.39 ± 0.84 points; and comprehension and judgment was 1.90 ± 0.40 points. The median MMSE-K score of the patients with AD was 16.50 (14.25–19.50); orientation 6.00 (4.50–6.75); memory registration 2.00 (1.00–3.00); memory recall 1.00 (0.25–1.75); attention 0.50 (0.00–1.00); language function 5.50 (5.00–6.75); and understanding and judgment 2.00 (2.00–2.00) points. The average MMSE-K score of patients with MCI was 23.35 ± 4.43; orientation 8.00 (6.00–10.00); memory registration 3.00 (3.00–3.00); memory recall 1.00 (0.00–2.50); attention 4.00 (1.00–5.00); language function 7.00 (6.00–7.00); and understanding and judgment 2.00 (2.00–2.00). The median MMSE-K score of patients with SCD was 26.00 (25.25–28.28); orientation was 9.50 (9.00–10.00); memory registration 3.00 (3.00–3.00); memory recall 2.50 (0.50–3.00); attention concentration score was 3.50 (2.25–4.75); language function was 6.50 (6.00–7.00); and understanding and judgment was 2.00 (2.00–2.00). The median MMSE-K score of the VD group was 26.00 (21.75–29.00); orientation 10.00 (8.50–10.00); memory registration 3.00 (3.00–3.00); memory recall 1.50 (0.75–2.00); attention scores were 4.00 (1.00–5.00); 7.00 (6.25–7.00) points for language function; and 2.00 (2.00–2.00) points for understanding and judgment (Table 2).

### 3.2 Primary index

#### 3.2.1 MMSE-K score analysis

The total MMSE-K score was significantly different from that the initial evaluation (p = 0.021 and p = 0.041, respectively). There was also a significant increase in the scores over time (p = 0.007).

Looking at the detailed items, the orientation score showed a significant difference after three months (p = 0.043), and attention and calculation scores increased significantly over time (p = 0.024). However, no significant changes were observed over time in orientation, memory registration, memory recall, language, or comprehension and judgment (Table 3).

**Table 3:** Score change in MMSE-K of total participants at baseline and 3, 6, and 9 months follow-up.

	Baseline	3 Months		6 Months		9 Months		Time Effect
		Score	P^†^	Score	P^†^	Score	P^†^	P^¶^
MMSE-K Total score	23.32 ± 4.66	24.58 ± 4.75	0.021*	24.32 ± 5.27	0.309	24.71 ± 5.10	0.041*	0.007*
Orientation	8.06 ± 2.00	8.58 ± 1.86	0.043*	8.48 ± 1.98	0.647	8.55 ± 1.95	0.421	0.092
Registration	2.74 ± 0.68	2.81 ± 4.78	1.000	2.84 ± 0.45	1.000	2.77 ± 0.56	1.000	0.504
Recall	1.35 ± 1.14	1.58 ± 1.20	1.000	1.52 ± 1.34	1.000	1.52 ± 1.23	1.000	0.593
Attention & Calculation	2.90 ± 1.92	3.29 ± 1.85	0.261	3.29 ± 1.94	0.378	3.52 ± 1.95	0.117	0.024*
Language	6.39 ± 0.84	6.45 ± 0.96	1.000	6.32 ± 1.01	1.000	6.48 ± 0.96	1.000	0.597
Comprehension & Judgment	1.90 ± 0.40	1.90 ± 0.40	>0.99	1.90 ± 0.40	>0.99	1.90 ± 0.40	>0.99	>0.99

MMSE-K, Korean version of Mini-Mental State Examination.

Values are presented as mean ± SD.

P*<0.05.

†: p-value for comparison by post-hoc analysis by Repeated Measure ANOVA with Bonferroni Correction.

¶: p-value for comparison by Repeated Measures ANOVA.

### 3.3 Secondary index

#### 3.3.1 Changes in MMSE-K scores in patients with AD

No significant change over time was observed in the total MMSE-K score of the AD group and all sub-items; however, the score increased or was maintained at the 9-month follow-up (Table 4).

**Table 4:** Score change in MMSE-K of AD group at baseline and 3, 6, and 9 months follow-up.

	Baseline	3 Months		6 Months		9 Months		Time Effect
		Score	P^†^	Score	P^†^	Score	P^†^	P^¶^
MMSE-K Total score	16.50 (14.25–19.50)	17.50 (14.25–20.75)	0.180	18.50 (14.00–20.00)	0.276	19.50 (14.50–20.00)	0.285	0.624
Orientation	6.00 (4.50–6.75)	7.00 (4.75–8.50)	0.180	7.00 (7.00–8.50)	0.102	7.00 (7.00–8.50)	0.102	0.066
Registration	2.00 (1.00–3.00)	2.50 (1.25–3.00)	0.317	3.00 (1.50–3.00)	0.317	2.00 (1.00–3.00)	1.0	0.392
Recall	1.00 (0.25–1.75)	0.50 (0.00–1.75)	0.317	0.00 (0.00–1.50)	0.157	1.00 (0.00–2.75)	0.564	0.392
Attention& Calculation	0.50 (0.00–1.00)	1.00 (0.25–1.00)	0.317	0.50 (0.00–1.00)	1.0	0.50 (0.00–1.00)	1.0	0.733
Language	5.50 (5.00–6.75)	5.50 (3.50–6.75)	0.414	5.00 (3.25–6.00)	0.102	5.50 (3.50–6.00)	0.257	0.421
Comprehension & Judgment	2.00 (2.00–2.00)	2.00 (2.00–2.00)	>0.9	2.00 (2.00–2.00)	>0.9	2.00 (2.00–2.00)	>0.9	>0.9

MMSE-K, Korean version of Mini-Mental State Examination; AD, Alzheimer's Disease.

Values are presented as median (Q1-Q3).

P*<0.05.

†: p-value for comparison by post-hoc analysis by Wilcoxon signed-rank test.

¶: p-value for comparison by Friedman's test.

#### 3.3.2 Changes in MMSE-K scores in patients with MCI

No significant change with time was observed in the total MMSE-K score of the MCI and all sub-items; however, the score increased or was maintained at the 9-month follow-up (Table 5).

**Table 5:** Score change in MMSE-K of MCI group at baseline and 3, 6, and 9 months follow-up.

	Baseline	3 Months		6 Months		9 Months		Time Effect
		Score	P^†^	Score	P^†^	Score	P^†^	P^¶^
MMSE-K Total score	23.35 ± 4.43	26.00 (22.50–28.50)	0.058	25.00 (22.50–28.00)	0.291	25.00 (22.50–28.50)	0.239	0.408
Orientation	8.00 (6.00–10.00)	9.00 (7.50–10.00)	0.058	9.00 (6.00–10.00)	0.341	9.00 (6.00–10.00)	0.437	0.158
Registration	3.00 (3.00–3.00)	3.00 (3.00–3.00)	0.414	3.00 (3.00–3.00)	0.414	3.00 (3.00–3.00)	0.414	0.801
Recall	1.00 (0.00–2.50)	2.00 (0.00–3.00)	0.618	1.00 (0.00–2.50)	1.00	1.00 (0.00–2.00)	0.836	0.838
Attention & Calculation	4.00 (1.00–5.00)	5.00 (3.00–5.00)	0.072	5.00 (2.00–5.00)	0.202	5.00 (2.00–5.00)	0.084	0.196
Language	7.00 (6.00–7.00)	7.00 (6.00–7.00)	0.739	7.00 (6.00–7.00)	0.739	7.00 (6.00–7.00)	0.589	0.557
Comprehension & Judgment	2.00 (2.00–2.00)	2.00 (2.00–2.00)	>0.9	2.00 (2.00–2.00)	>0.9	2.00 (2.00–2.00)	>0.9	>0.9

MMSE-K, Korean version of Mini-Mental State Examination; MCI, mild cognitive impairment.

Values are presented as mean ± SD, median (Q1-Q3).

P*<0.05.

†: p-value for comparison by post-hoc analysis by Wilcoxon-signed rank test.

¶: p-value for comparison by Friedman's test.

#### 3.3.3 Changes in MMSE-K scores in patients with SCD

No significant change over time was observed in the total MMSE-K score of the SCD group and all sub-items; however, the score increased or was maintained at the 9-month follow-up (Table 6).

**Table 6:** Score change in MMSE-K of SCD group at baseline and 3, 6, and 9 months follow-up.

	Baseline	3 Months		6 Months		9 Months		Time Effect
		Score	P^†^	Score	P^†^	Score	P^†^	P^¶^
MMSE-K Total score	26.00 (25.25–28.25)	26.50 (25.25–29.25)	0.157	27.50 (25.25–29.75)	0.180	29.00 (26.75–29.75)	0.109	0.223
Orientation	9.50 (9.00–10.00)	9.50 (9.00–10.00)	1.000	10.00 (9.25–10.00)	0.317	10.00 (9.25–10.00)	0.564	0.753
Registration	3.00 (3.00–3.00)	3.00 (3.00–3.00)	>0.99	3.00 (3.00–3.00)	>0.99	3.00 (3.00–3.00)	>0.99	>0.99
Recall	2.50 (0.50–3.00)	2.50 (0.50–3.00)	1.000	3.00 (0.75–3.00)	0.317	2.50 (0.50–3.00)	1.000	0.733
Attention & Calculation	3.50 (2.25–4.75)	4.00 (2.25–5.00)	0.317	4.50 (2.50–5.00)	0.157	5.00 (4.25–5.00)	0.102	0.112
Language	6.50 (6.00–7.00)	7.00 (6.25–7.00)	0.317	6.50 (6.00–7.00)	1.000	7.00 (7.00–7.00)	0.157	0.194
Comprehension & Judgment	2.00 (2.00–2.00)	2.00 (2.00–2.00)	>0.99	2.00 (2.00–2.00)	>0.99	2.00 (2.00–2.00)	>0.99	>0.99

MMSE-K, Korean version of Mini-Mental State Examination; SCD, subjective cognitive decline.

Values are presented as median (Q1-Q3).

P*<0.05.

†: p-value for comparison by post-hoc analysis by Wilcoxon signed-rank test.

¶: p-value for comparison by Friedman's test.

#### 3.3.4 Changes in MMSE-K scores in patients with VD

The total MMSE-K score in the VD group showed a statistically significant increase over time (p = 0.015). At a single time point, there was a significant increase compared with the initial evaluation after 9 months (p = 0.026). However, there was no significant increase in the number of detailed items over time (Table 7).

**Table 7:** Score change in MMSE-K of VD group at baseline and 3, 6, and 9 months follow-up.

	Baseline	3 Months		6 Months		9 Months		Time Effect
		Score	P^†^	Score	P^†^	Score	P^†^	P^¶^
MMSE-K Total score	26.00 (21.75–29.00)	27.50 (24.75–29.00)	0.066	29.00 (23.50–30.00)	0.171	28.50 (25.50–30.00)	0.026*	0.015*
Orientation	10.00 (8.50–10.00)	10.00 (9.75–10.00)	0.180	10.00 (9.00–10.00)	1.000	10.00 (9.75–10.00)	0.180	0.284
Registration	3.00 (3.00–3.00)	3.00 (2.75–3.00)	0.317	3.00 (2.75–3.00)	0.317	3.00 (2.75–3.00)	0.317	0.392
Recall	1.50 (0.75–2.00)	2.00 (1.75–3.00)	0.059	3.00 (1.50–3.00)	0.063	2.50 (1.00–3.00)	0.059	0.097
Attention & Calculation	4.00 (1.00–5.00)	3.50 (1.75–5.00)	1.000	4.50 (2.00–5.00)	0.414	4.50 (3.50–5.00)	0.461	0.915
Language	7.00 (6.25–7.00)	7.00 (6.75–7.00)	0.317	7.00 (6.00–7.00)	0.655	7.00 (6.75–7.00)	0.317	0.572
Comprehension & Judgment	2.00 (2.00–2.00)	2.00 (2.00–2.00)	>0.99	2.00 (2.00–2.00)	>0.99	2.00 (2.00–2.00)	>0.99	>0.99

MMSE-K, Korean version of Mini-Mental State Examination; VD, Vascular dementia.

Values are presented as median (Q1-Q3).

P*<0.05.

†: p-value for comparison by post-hoc analysis by Wilcoxon signed-rank test.

¶: p-value for comparison by Friedman's test.

#### 3.3.5 S-GDS score analysis

S-GDS scores decreased significantly over time (p = 0.006). Compared with the initial evaluation, significant improvements were observed at 3 and 6 months (p = 0.038 and p = 0.023, respectively) (Table 8).

**Table 8:** Score change in S-GDS of all participants at baseline and 3 and 6 months follow-up.

	Baseline	3 Months		6 Months		Time Effect
		Score	P^†^	Score	P^†^	P^¶^
S-GDS (n = 9)^§^	2.00 (1.00–3.00)	1.00 (1.00–2.00)	0.038*	1.00 (0.50–2.00)	0.023*	0.006*

S-GDS, Short version of Geriatric Depression Scale.

Values are presented as median (Q1-Q3).

P*<0.05.

†: p-value for comparison by post-hoc analysis by Wilcoxon signed-rank test.

¶: p-value for comparison by Friedman's test.

§: 6 participants for MCI, 1 participant for SCD, 2 participants for VD.

## 4 Discussion

In terms of the sex ratio 29.0 % more males than females had dementia in Korea as announced by the Central Dementia Center [1]. However, the male sex ratio was 75.0 % in the SCD group because male patients of working age did not visit the clinic with mild symptoms.

In this study, the MMSE-K was selected as the primary index. In a previous KGT study, the MMSE score worsened in the group taking donepezil alone for Alzheimer's disease, and was maintained in the donepezil and KGT combination group [11]. In the group taking KGT, the MMSE score significantly increased to 1.65 ± 0.53 (p = 0.015) [12]. Not only did the total MMSE-K score increase significantly in this study, but there was also a significant time effect at three and nine months after starting KGT. In addition, a significant effect was observed at three months compared with the initial evaluation of the orientation item among the detailed items, and a significant time effect was also observed in the attention and calculation items. This is consistent with the results of a previous study that showed that orientation and attention significantly improved in the KGT group [12].

In this study, no significant changes in the MMSE-K scores were observed in the AD, MCI, and SCD groups. First, in the AD group, it can be estimated that the disease did not progress significantly, as no significant deterioration in the MMSE-K was observed with time, even after nine months. The score did not change in the mild cognitive impairment group because the sensitivity of the MMSE-K evaluation tool in detecting mild cognitive impairment is low. There were also no significant change in the scores in the SCD group. Similarly, the sensitivity of the MMSE-K for detecting MCI was low and it appeared to be in the normal category in the initial evaluation. This was not statistically significant, and the score increased or was maintained at the 9-month follow-up.

In contrast, in the VD group, there was not only a significant improvement in the total MMSE-K score at 9 months, but also a significant improvement over time. This is in contrast to the general stroke characteristic of natural improvement in general cognitive impairment up to 3 months after onset, followed by no significant improvement despite treatment.

Nine patients were evaluated using the S-GDS, including six with MCI, one with SCD, and two with VD. The number of patients included was small because this evaluation was added after 2021 because of the medical record analysis.

Although the S-GDS had a low score with an initial median of 2 points, it decreased significantly between 3 and 6 months. Behavioral symptoms, such as depression, affect cognitive deterioration [19]. Therefore, KGT is clinically prescribed for depression, and is considered to have potential for improving cognitive function and exacerbation factors.

A limitation of this study is that the subgroups of AD, MCI, SCD, and VD had a small number of samples, so that significant results may not be observed owing to low power. Additionally, the MMSE-K was used as a cognitive function-related evaluation tool. Although the MMSE-K has the advantages of being easy and taking a short time in the clinic, it has low sensitivity for detecting MCI and the disadvantage of a ceiling effect [20]. In future studies, it will be desirable to use additional evaluation tools, such as the Montreal Cognitive Assessment [21] in addition to the MMSE-K. Finally, because this was a retrospective study of outpatients, some evaluations were omitted, resulting in missing values. Acupuncture and moxibustion were combined, and patients taking decoctions were excluded.

The strength of this study is that, unlike previous studies on AD and MCI, the effect of KGT on cognitive impairment was confirmed through a retrospective chart review based on the records of treatment and evaluation in a clinical setting. In addition, unlike previous studies, the continuous dosing period was long, and it was meaningful to confirm the trend over time by conducting evaluations several times at 3-month intervals.

## 5 Conclusion

In conclusion, the administration of KGT to patients with cognitive impairment confirmed the possibility that it could be effective for treating various symptoms such as cognitive function and depression in patients who complain of cognitive impairment in the clinical field. Further large-scale, multicenter, retrospective chart reviews are needed to confirm these conclusions.

## Ethics approval

All procedures in this retrospective study were conducted in compliance with the ethical regulations of the Declaration of Helsinki and were approved by the Institutional Review Board of Kyung Hee University Hospital at Gangdong (IRB approval number: KHNMCOH 2022-03-008).

## Data availability statement

Data will be made available on request.

## CRediT authorship contribution statement

**Tae-Bin Yim:** Data curation, Formal analysis, Methodology, Writing – original draft, Writing – review & editing. **Gyu-Ri Jeon:** Data curation. **Hye-Jin Lee:** Data curation. **Kyeong-Hwa Lee:** Data curation. **Hye-Min Heo:** Data curation. **Han-Gyul Lee:** Supervision. **Seungwon Kwon:** Conceptualization. **Seung-Yeon Cho:** Supervision. **Seong-Uk Park:** Supervision, Validation. **Woo-Sang Jung:** Validation. **Sang-Kwan Moon:** Supervision. **Chang-Nam Ko:** Conceptualization. **Jung-Mi Park:** Methodology, Supervision, Validation.

## Declaration of competing interest

The authors declare that they have no known competing financial interests or personal relationships that could have appeared to influence the work reported in this paper.
